# *N*^*6*^-methyl-adenosine level in *Nicotiana tabacum* is associated with tobacco mosaic virus

**DOI:** 10.1186/s12985-018-0997-4

**Published:** 2018-05-16

**Authors:** Zhurui Li, Jing Shi, Lu Yu, Xiaozhen Zhao, Longlu Ran, Deyu Hu, Baoan Song

**Affiliations:** State Key Laboratory Breeding Base of Green Pesticide and Agricultural Bioengineering/Key Laboratory of Green Pesticide and Agricultural Bioengineering, Ministry of Education, Guizhou University, Guiyang, 550025 China

**Keywords:** *N. tabacum*, TMV, m^6^A, UHPLC−HR − MS/MS, RT-qPCR

## Abstract

**Background:**

*N*^*6*^-methyl-adenosine (m^6^A) is a prevalent RNA modification in many species. Abnormal m^6^A methylation levels can lead to RNA dysfunction and can cause diseases. Tobacco mosaic virus (TMV) is one of the most devastating viruses for agricultural plants. It has many hosts, particularly including tobacco and other members the family Solanaceae. However, it remains unclear whether the abnormal growth induced by TMV is associated with the m^6^A level.

**Methods:**

A rapid and accurate analytical method using ultra-high-performance liquid chromatography coupled with high-resolution tandem mass spectrometry (UHPLC−HR − MS/MS) was developed to analyse the adenosine (A), cytidine (C), guanosine (G), uridine (U), and m^6^A contents in the tobacco leaf, and the m^6^A/G ratio was used to evaluate the m^6^A level. Subsequent protein sequence alignments were used to find the potential methylases and demethylases in *Nicotiana tabacum* (*N. tabacum*)*.* Finally, reverse transcription quantitative real-time polymerase chain reaction (RT-qPCR) was used to analyse the gene expression levels of the potential methylases and demethylases in the *N. tabacum* leaf.

**Results:**

The results showed that TMV reduced the m^6^A level. Moreover, protein sequence alignments revealed partial homology among human ALKBH5, *Arabidopsis* (NP_001031793), and *Nicotiana sylvestris* (XP_009800010). The gene expression level of the potential demethylase XM_009801708 increased at 14 and 21 days in *N. tabacum* infected with TMV, whereas all of the potential methylases decreased.

**Conclusions:**

The reversible m^6^A modification in *N. tabacum* mRNA might represent a novel epigenetic mechanism involved in TMV.

**Electronic supplementary material:**

The online version of this article (10.1186/s12985-018-0997-4) contains supplementary material, which is available to authorized users.

## Background

M^6^A is a prevalent RNA modification in species such as viruses, bacteria [1], yeasts [2], plants [3], and mammals [4, 5]. The modification of m^6^A is mediated by *N*^*6*^-adenosine methyltransferase complexes such as 70 kD SAM (*S*-adenosylmethionine)-binding subunit methyltransferase-like 3 (METTL3, also called MT-A70), methyltransferase-like 14 (METTL14), and Wilms tumour 1-associated protein (WTAP) [6, 7]. METTL3 and METTL14 serve as the catalytic subunits, and WTAP serves as the regulatory subunit. The discovery of m^6^A demethylases of FTO (fat mass and obesity-associated protein) [8] and ALKBH5 [9] reveals that RNA methylation is reversible and can dynamically the mRNA metabolism. m^6^A methylation plays a broad and important role in the functional interplay among m^6^A methylases, demethylases, and m^6^A binding proteins [3]. Abnormal m^6^A methylation levels, which are induced by defects in any factor in this network, can lead to RNA dysfunction and can cause diseases [10–14]. Zhong et al. [11] report that the inactivation of mRNA adenosine methylase (MTA) prevents the progression of the developing embryo from entering the globular stage, leading to an embryo-lethal phenotype with seed arrestment in plants. Bodi et al. [15] report that the reduced expression of MTA in *Arabidopsis thaliana* (*A. thaliana*) reduces the m^6^A level in mRNA and induces abnormal growth, including a reduction of apical dominance, abnormal organ definition, and an increase in trichome branching. Shen et al. [16] show that FIP37 is a core component of the m^6^A methyltransferase complex and determines Arabidopsis m6A mRNA modification pattern. FIP37 mediates m6A modification of key shoot meristem regulator mRNAs and, through the control of their stability, prevents overproliferation of the shoot apical meristem that produces all aerial organs. However, it remains unclear whether the abnormal growth induced by plant viruses is associated with the m^6^A levels.

*Nicotiana tabacum (N. tabacum)* is the most commonly grown of all plants in the *Nicotiana* genus. The three most commonly used tobacco types are Flue-Cured (or Virginia), Burley and Oriental. *N. tabacum* K326 belongs to type of Flue-Cured. Tobacco is used as a model for plant disease susceptibility, which it shares with other *Solanaceae* plants including potato, tomato and pepper. Recently research confirmed that *Nicotiana sylvestris* and *Nicotiana tomentosiformis* are the most likely progenitors of *N. tabacum.* [17]. Tobacco mosaic virus (TMV) is a positive-sense single stranded RNA virus, genus tobamovirus. It has a wide host range, including vegetables, ornamentals, legumes, and other important crops [18]. Although TMV has been studied for more than 110 years [19], the association between m^6^A and TMV has not been reported.

The traditional methods for studying RNA modifications are ^32^P-labelling, 2D thin-layer chromatography [11, 20, 21], dot-blot [8], high-performance liquid chromatography (HPLC) [10], and capillary electrophoresis coupled with laser-induced fluorescence detection [22]. However, these methods are tedious, semi-quantitative, and low-throughput, and they require a large amount of RNA. HPLC coupled with a triple-quadruple mass spectrometry using external standards was recently used to measure the m^6^A levels in mRNA [9, 23], other RNA modifications in tRNA and small RNA, and a femtomole level of sensitivity was achieved [24]. Furthermore, a method of stable isotope-labelled internal standards was used to measure these post-transcriptionally modified nucleosides in the cellular RNA species [25]. Although this method is more accurate than a triple-quadruple mass spectrometer using external standards, the production of stable isotope-labelled compounds is complicated and time-consuming. Therefore, a rapid and accurate analytical method is required to better understand the relationship between TMV and m^6^A methylation.

A rapid method to determine the levels of adenosine (A), cytidine (C), guanosine (G), uridine (U), and m^6^A in mRNA in *Nicotiana tabacum* (*N. tabacum*) using ultra-high-performance liquid chromatography coupled with high-resolution tandem mass spectrometry (UHPLC−HR − MS/MS) was developed in this study. This assay was used to determine the effect of TMV on m^6^A mRNA methylation in *N. tabacum*. Subsequent protein sequence alignments were applied to find the potential methylases and demethylases in *N. tabacum.* Finally, RT − qPCR was used to investigate the gene expression of potential methylases and demethylases in *N. tabacum*.

## Methods

### Plant materials and growth conditions

*N. tabacum* K326 seeds were purchased from the Guizhou Academy of Tobacco Science. TMV was obtained from the Wuhan Institute of Virology at the Chinese Academy of Sciences, and its source was preserved in a *N. tabacum* K326 plant over an extended period. The extraction and purification of TMV were conducted as previously described by Gooding and Iwata [26]. *N. tabacum* K326 seeds were grown in a disease-free greenhouse, and 12-week-old plants were used for the study. The temperature and relative humidity levels in the greenhouse were maintained close to 25 °C and 60%, respectively.

The leaf samples were divided into two groups: a control group of healthy *N. tabacum* leaves and a treatment group of *N. tabacum* leaves infected with TMV. *N. tabacum* were mechanically inoculated by dusting carborundum on the leaves, wetting a brush with the inoculum and rubbing the leaves of selected plants. The inoculated leaves were rinsed with sterile distilled water 30 min after inoculation. The controls were prepared in a similar fashion using sterile PBS [27]. PCR was used to detect whether the *N. tabacum* infected TMV (see Additional file 1). The leaf samples were collected at 3, 5, 7, 10, 14, and 21 days after inoculation with TMV, removed from the main leaf vein, frozen in liquid nitrogen, and stored at − 80 °C before use. The collected leaf samples in the treatment group were not inoculated by TMV directly. Each experiment was repeated three times.

### Sample preparation

To prepare mRNA from *N. tabacum* leaves, 0.50 g of *N. tabacum* leaves was prepared, and the total RNA was extracted using TRIzol reagent (Takara Biotechnology Co. Ltd., Otsu, Japan) according to the manufacturer’s protocol. RNA pellets were dissolved in 200 μL of 1% diethylpyrocarbonate H_2_O (DEPC-water), and the quality was tested using an ASP-3700 ultraviolet/visible (UV-vis) spectrophotometer (Xiamen biotech co., LTD, Xiamen, China) and gel electrophoresis. The total mRNA was isolated using poly(dT) oligo magnetic beads, followed by rRNA depletion. The mRNA concentrations and qualities were measured using UV spectrophotometry.

To digest mRNAs to nucleosides, 0.5 unit of nuclease P1 (Sigma-Aldrich, St. Louis, MO) and 2.5 μL of 1 M NH_4_Ac (pH = 5.3) were added to 50 ng of mRNA. DEPC-water was added to obtain a 20 μL volume. The reaction mixture was incubated at 42 °C for 8 h. Before adding 1 unit of alkaline phosphatase (Sigma-Aldrich, St. Louis, MO, USA), 2.5 μL of NH_4_HCO_3_ was added. After digestion at 37 °C for 8 h, the resulting digestion mixture was filtered through a 0.22-μm nylon syringe filter for UHPLC−HR − MS/MS analysis.

### Condition of UHPLC−HR − MS/MS

UHPLC−HR − MS/MS analyses of A, C, G, U, and m^6^A were conducted on an UltiMate 3000 UHPLC coupled with a Q Exactive high resolution mass spectrometer (Thermo Fisher Scientific, Foster City, CA, USA).

Chromatographic separation of A, C, G, U, and m^6^A was performed on an UltiMate 3000 UHPLC system, a binary solvent manager, a column oven, a solvent degasser, and an autosampler equipped with an Agilent ZORBOX SB-Aq column (4.6 mm × 50 mm, 1.8 μm particle size; Santa Clara, CA, USA). Solutions of 0.1% (*v*/v) formic acid in water (solution A) and 0.1% (v/v) formic acid in methanol (solution B) were used as the mobile phases. The nucleosides were separated using a gradient of 20% B for 1 min, 20–40% B for 2 min, 40% B for 3 min, 40–20% B for 0.01 min, and 20% B for 2 min. The flow rate was 0.3 mL/min. The column oven temperature was maintained at 40 °C to reduce the viscosity, and the temperature of the sample vial holder was set at 4 °C. The injected sample volume was maintained at 2 μL. The target compounds were all eluted within 8 min.

A Q Exactive high-resolution mass spectrometer equipped with an electrospray ionization source was used to quantify the tested analytes. Nitrogen was used as the sheath gas and aux gas. The electrospray ionization source-dependent parameter settings were as follows: sheath gas flow rate, 35 psi (nitrogen); aux gas flow rate, 8 psi (nitrogen); spray voltage, 3.5 kV; capillary temperature, 320 °C; aux gas heater temperature, 300 °C; and S-lens RF level, 50. The target selected ion monitoring (SIM) in positive mode was used for the quantitative determination of A, C, G, U, and m^6^A. The resolution of the mass spectrometer was 70,000. *m/z* values of 245.07681 (U), 282.11968 (m^6^A), 284.09895 (G), 244.09289 (C), and 268.10403 (A) were used for the qualitative detection and quantification.

### Method validation

To ensure that the method was accurate and reliable, the method was validated to evaluate its performance according to the Food and Drug Administration Guidance for Industry Bioanalytical Method Validation [28]. The validation parameters included the linear range, limit of detection (LOD), limit of quantification (LOQ), precision, and accuracy.

The LOD and LOQ were 3 and 10, respectively, as determined using S/N. The intra- and inter-day accuracy and precision were assessed by analysing the standard solutions of ribonucleosides at three different concentrations. The inter-day results were obtained from analyses of five replicates conducted on 3 separate days. Accuracy and precision were expressed as the percent recovery and relative standard deviation (RSD), respectively. The stabilities of analytes present in mRNA were evaluated after three cycles of freeze (at − 20 °C for 24 h) and thaw (at room temperature). The percent recovery was evaluated by comparing the levels before and after the freeze-and-thaw cycles, and the RSD was calculated based on the present recovery obtained from three aliquots of RNA samples.

### Protein sequence alignment

To discover the potential methylases and demethylases in *N. tabacum* and investigate conservation of a family of ALKB in diverse species, amino acid sequences of human ALKBH5, closely related proteins from *A. thaliana*, and *Nicotiana sylvestris* (*N. sylvestris*) were identified using database searches and aligned using AlignX (a component of Vector NTI Suite 11.0 software, InforMax Inc., Rockville, MD, USA), and the protein sequences were obtained from NCBI information (https://www.ncbi.nlm.nih.gov/pmc/).

### Method of RT–qPCR

To investigate the reason for the m^6^A level decrease, RT − qPCR was used to study the gene expression level of the potential methylases and demethylases in *N. tabacum*.

The gene sequences analysed by RT-qPCR in *N. tabacum* were obtained from the BLAST searches in NCBI (https://www.ncbi.nlm.nih.gov/pmc/) and compared to the methylase and potential demethylase of *A. thaliana.* The detailed RT-qPCR operation was performed as described below. First, 0.15 g of *N. tabacum* leaves was prepared, and the total RNA was extracted using TRIzol reagent according to the manufacturer’s protocol. RNA pellets were dissolved in 30 μL of RNA-free water with concentration and purity determined by an ASP-3700 UV spectrophotometer. Reverse transcription (RT) was performed to synthesize cDNA using the PrimeScript™ RT reagent Kit (Takara Biotechnology Co. Ltd., Otsu, Shiga, Japan) in C1000™ Thermal Cycler (Bio-Rad, Hercules, CA, USA). The qPCR primers were designed using the online NCBI sequence information. The target genes were XM_009766347, XM_009766348, XM_009775897, XM_009770927, XM_009801708, and XM_009801663, and the reference gene was *β*-actin. The primers of target genes were described in [see Additional file 1: Table S1]. Plasmid transformation and cloning was conducted using a pUCm-T DNA Cloning Vector Kit (Sangon Biotech, Shanghai, China). The sequences of target genes were validated by sequencing using a pUCm-T DNA Cloning Vector Kit, and the sequences agreed with the NCBI sequence information. qPCR analysis was conducted using SYBR Green technology with the SYBR® Premix Ex Taq™ II (TliRNaseH Plus) (Takara Biotechnology Co. Ltd., Otsu, Shiga, Japan) in an iCycleriQ™ instrument according to the manufacturer’s instructions (Bio-Rad, Hercules, CA, USA). The optimized RT − qPCR parameters included the annealing temperature, number of cycles, and cDNA concentration. The thermal cycling profile for qPCR amplifications was as follows: 95 °C for 30 s, followed by 35 cycles at 95 °C for 30 s, 58 °C for 30 s, and 72 °C for 30 s. The relative quantification of the expression levels of selected genes was performed using the 2^-ΔΔCt^ method.

### Statistical analysis

Data were expressed as the means ± standard deviation (SD) of at least triplicate assays. Statistical differences of the data were evaluated by using IBM SPSS Statistics 19. The differences between the measurement data were analysed using the *t*-test, and differences were considered significant at *P* ≤ 0.05 or *P* ≤ 0.01.

## Results

### Optimization of MS/MS

The performances of three types of columns, including the Agilent ZORBOX SB-Aq column (4.6 mm × 50 mm, 1.8 μm particle size, Santa Clara, CA, USA), Thermo Scientific Hypersil GOLD column (2.1 mm × 150 mm, 1.9 μm particle size, Foster City, CA, USA), and Phenomenex Desc. Kinetex C18 (2.1 mm × 100 mm, 2.6 μm particle size, Los Angeles, CA, USA) were tested. The other two columns required a longer running time than did the Agilent ZORBOX SB-Aq column. Thus, A, C, G, U, and m^6^A could be well-separated on Agilent ZORBOX SB-Aq column in 8 min.

The linearity, LOD, and LOQ were obtained using the peak areas of the product ion with the target SIM mode. As shown in Table 1, the linearity was evaluated by preparing different calibration curves (A, C, G, U, and m^6^A). The regression equations and determination coefficients (*R*^2^) of all the standard solution curves indicated that all the target compounds showed excellent linearity (*R*^2^ ≥ 0.9989 in all cases) [see Additional file 1: Figures S1–S5]. The LODs for A, C, G, U, and m^6^A were 0.0010, 0.0045, 0.0030, 0.0476, and 0.0003 μM, respectively. The LOQs for A, C, G, U, and m^6^A were 0.0032, 0.0148, 0.0100, 0.1587, and 0.0009 μM, respectively.

**Table 1:** Calibration equations, determination coefficients (*R*^2^), LODs, and LOQs of A, C, G, U, and m^6^A in the solvent

Analyte	Calibration equations	*R* ^2^	LOQ (μM)	LOD (μM)
C	*y* = 6 × 10^6^*x* – 3 × 10^6^	0.9994	0.0148	0.0045
U	*y* = 1.08029 × 10^5^*x* − 2.52572 × 10^5^	0.9994	0.1587	0.0476
G	*y* = 8 × 10^6^*x* + 4.97703 × 10^5^	0.9999	0.0100	0.0030
A	*y* = 1 × 10^8^*x* + 7 × 10^6^	0.9989	0.0032	0.0010
M^6^A	*y* = 2.01953 × 10^5^*x* + 3.11036 × 10^5^	0.9999	0.0009	0.0003

The intra- and inter-day accuracy and precision were evaluated by analysing three different concentrations of standard solutions of nucleosides. As shown in Table 2, the method was satisfactorily accurate and precise in measuring the nucleosides. The intra-day recoveries for the five ribonucleosides ranged from 86.9 to 114.8%, and the RSDs for all the analytes ranged from 1.2 to 9.5%. We further investigated the stabilities of analytes present in mRNA after three cycles of freeze (at − 20 °C for 24 h) and thaw (to room temperature). The analytes were reasonably stable under freezing/thaw conditions, as reflected by the inter-day recovery of 89.3%–106.3% and the RSD range of 1.0 to 9.0%.

**Table 2:** Precision and accuracy for the measurements of A, C, G, U, and m^6^A from three validation runs (2 μL of aliquot was injected in each run)

Analyte	Concentration (μM)	Intra-day		Inter-day	
		Mean recovery± SD (%)	RSD (%)	Mean recovery± SD (%)	RSD (%)
A	0.100	87.7 ± 1.1	1.2	89.3 ± 0.9	1.0
	1.000	86.9 ± 4.2	4.9	97.4 ± 6.4	6.5
	2.500	94.5 ± 0.7	0.8	90.6 ± 6.9	7.6
G	0.250	87.2 ± 3.5	4.0	94.8 ± 7.6	8.0
	2.500	89.9 ± 0.4	0.5	99.6 ± 2.6	2.7
	12.500	102.5 ± 1.9	1.9	106.3 ± 4.4	4.1
U	5.000	96.6 ± 4.3	4.3	100.3 ± 2.1	2.1
	25.000	87.6 ± 9.5	9.5	100.3 ± 4.3	4.3
	250.000	96.9 ± 6.9	6.9	96.5 ± 2.0	2.0
C	0.500	102.5 ± 0.4	0.3	99.2 ± 4.6	4.6
	2.500	101.5 ± 8.3	8.2	98.7 ± 7.3	7.4
	25.000	114.8 ± 5.7	4.9	90.0 ± 2.8	3.1
M^6^A	0.005	93.7 ± 2.7	2.9	91.2 ± 4.7	5.2
	0.025	90.9 ± 3.2	3.5	91.4 ± 8.2	9.0
	0.125	91.9 ± 2.7	2.9	95.1 ± 4.9	5.2

**Fig. 1:**
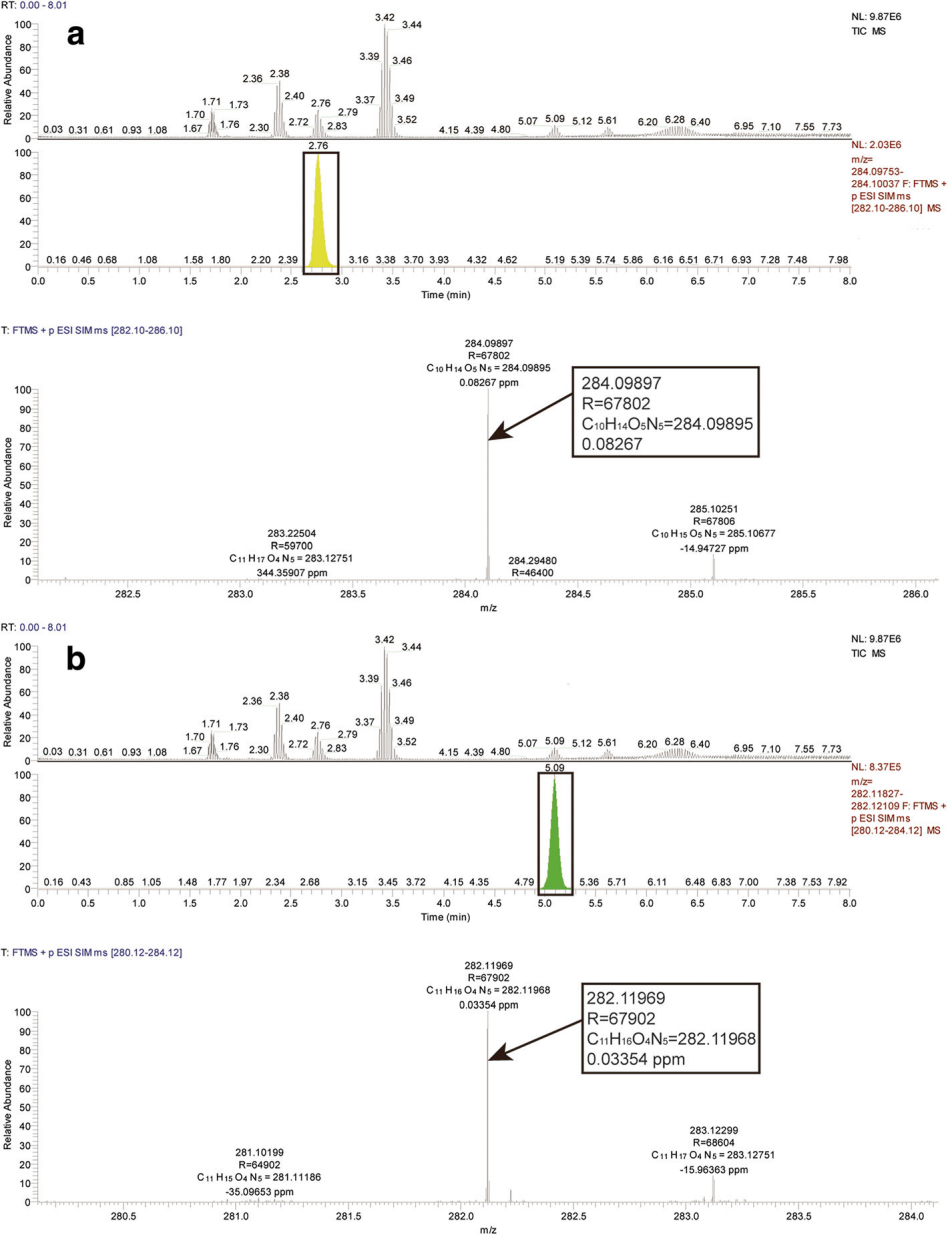
Representative UHPLC-HR-MS/MS results for G (**a**) and m^6^A (**b**) in *N. tabacum*. TIC MS shows the separated components. FTMS + p ESI SIM MS shows the chromatogram of objective component and its precise mass, resolution, and mass error

An UHPLC−HR − MS/MS method was developed for the accurate assessment of A, C, G, U, and m^6^A in mRNA isolated from *N. tabacum.* Figure 1 and [see Additional file 1: Figures S6–S8] showed the representative UHPLC−HR − MS/MS results for the quantifications of A, C, G, U, and m^6^A in *N. tabacum* with selected-ion chromatograms. As shown in Fig. 1 and [see Additional file 1: Figures S6–S8], the characteristic ions at m/z 282.11969, m/z 284.09897, m/z 268.10406, m/z 244.09282, and m/z 245.07687 were assigned to m^6^A [C_11_H_15_O_4_N_5_ + H] ^+^, G [C_11_H_15_O_5_N_5_ + H] ^+^, A [C_10_H_13_O_4_N_5_ + H] ^+^, C [C_9_H_13_O_5_N_3_ + H] ^+^, U [C_9_H_12_O_6_N_2_ + H] ^+^, respectively. All the mass errors of precursors were less than 0.25 ppm.

### TMV induced a decrease in m^6^A level in *N. tabacum*

**Fig. 2:**
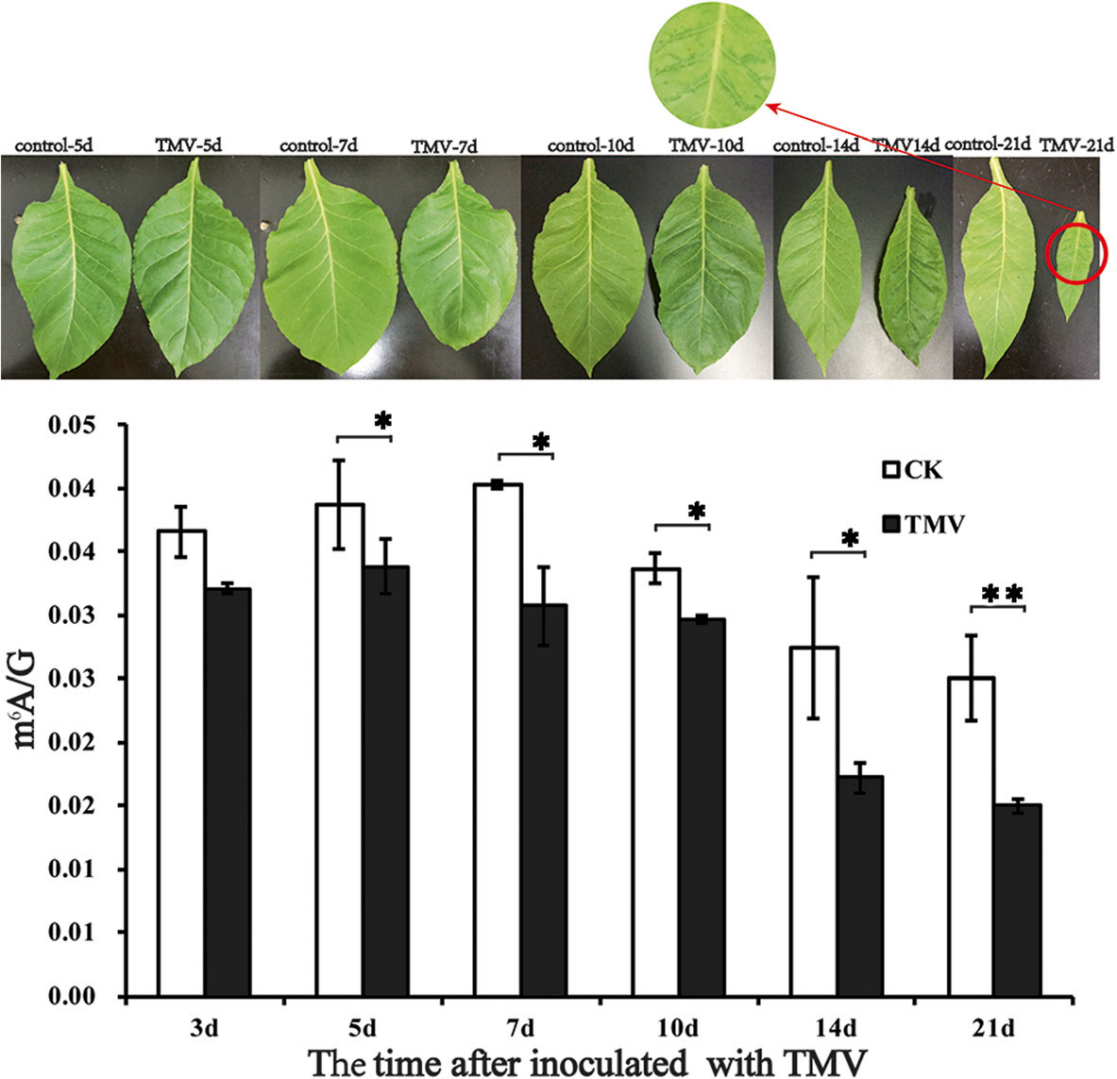
m^6^A levels after infection with TMV after 14 and 21 days compared with control. The tobacco leaves present clear dysmorphosis at day 14 and appear characteristically mosaic at day 21. Each experiment represents the mean ± SD of at least three duplicates (*n* = 3).^*^P ≤ 0.05, ^**^P ≤ 0.01 vs. control

In this study, the ratio of m^6^A/G in *N. tabacum* was recorded during TMV infection. TMV reduced the m^6^A level in *N. tabacum*. As shown in Fig. 2, the m^6^A methylation levels decreased by 12.30, 12.66, 23.82, 12.02, 37.34, and 40.00% for 3, 5, 7, 10, 14, and 21 days, respectively, compared to the control. The level of m^6^A decreased most significantly at 21 days after TMV infection. In addition, the tobacco leaves presented dysmorphosis as time went on, especially the occurring characteristic mosaic at 21 days. This finding suggested that m^6^A played an important role in the growth of *N. tabacum* and that TMV reduced the m^6^A level in *N. tabacum.*

### Conservation of a family of ALKB in diverse species

To analyse the conservation of ALKB gene family, we compared amino acid sequences from the ALKB family. The sequence alignment results showed that the potential methylases of *Nicotiana sylvestris* were methyltransferase-like protein 1 (LOC104232562) (XM_009785798), methyltransferase-like protein 1 (LOC104216329) (XM_009766348), and FKBP12-interacting protein of 37 kDa-like (LOC104224278) (XM_009775897). The potential demethylases of *Nicotiana sylvestris* were uncharacterized LOC104220112 (LOC104220112) (XM_009770927), uncharacterized LOC104245988 (LOC104245988) (XM_009801708), and uncharacterized LOC104245950 (LOC104245950) (XM_009801663).

**Fig. 3:**
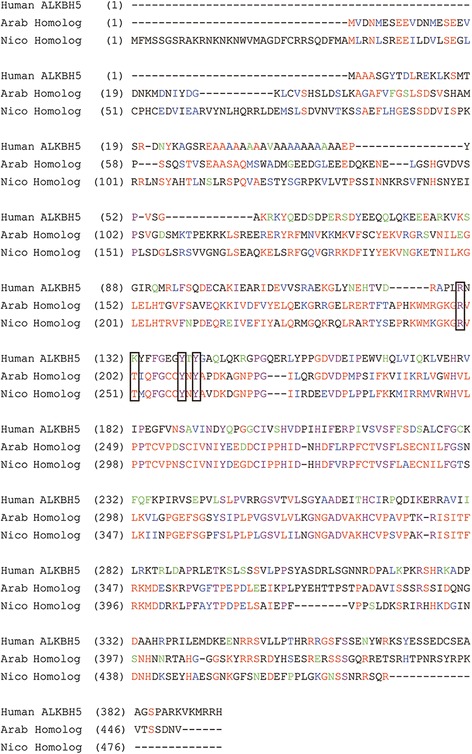
Conserved protein sequences of putative mRNA *N*^*6*^-methyl-adenosine demethylases. The red letters represent the consensus residues derived from the conserved amino acid at a given position. Identical residues are shown in purple, and blocks of similar residues are shown in blue. Weak similarities are depicted in green. The overall sequence identity was ~ 40% across all proteins, and the sequence similarity was almost 95% across this region

As shown in Fig. 3, database analysis and protein sequence alignments revealed partial homology between human ALKBH5 (NP_060228), *Arabidopsis* (NP_001031793, putative demethylase), and *N. sylvestris* (XP_009800010, putative demethylase, which was encoded by XM_009801708).

### ALKB family expression analysis

In the RT − qPCR experiments, the concentration and 260/280 and 260/230 ratios of RNA were 1244.9–2397.8 ng/μL, 1.92–2.28, and 1.54–2.26, respectively.

**Fig. 4:**
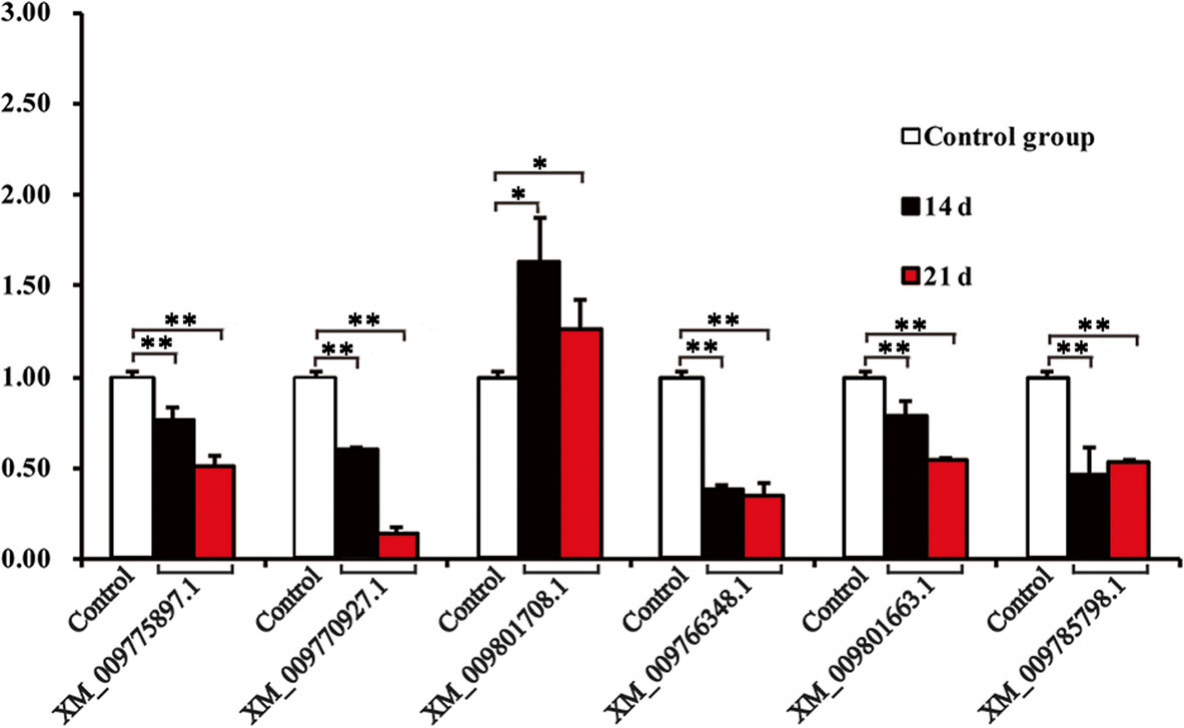
Gene expression levels of putative methylases and demethylases. The potential methylases are XM_009766347, XM_009766348, and XM_009775897. The potential demethylases are XM_009770927, XM_009801708, and XM_009801663 in *N. tabacum*.^*^P ≤ 0.05, ^**^P ≤ 0.01 vs. control

As shown in Fig. 4, the gene expression level of the XM_009801708 increased at 14 and 21 days, and the expression levels of the tested potential methylases decreased. These results suggested that XP_009800010 was the most likely demethylase of *N. tabacum*.

## Discussion

LC − MS/MS was successfully used to identify and quantify post-transcriptionally modified nucleosides in RNA [9, 23–25]. In this study, an UHPLC−HR − MS/MS method was developed to detect A, C, G, U, and m^6^A in mRNA that was isolated from *N. tabacum*. This method showed several advantages compared with previously reported methods. First, only 0.50 g of *N. tabacum* leaves was needed to prepare RNA because of the high sensitivity. Second, the analytes could be simultaneously detected by UHPLC−HR − MS/MS under the target SIM mode within 8 min, which significantly increased its efficiency compared with previous methods [8, 10, 23, 25, 29]. Moreover, SIM mode could avoid any interference from the matrix. Third, more accurate detection results could be obtained by UHPLC−HR − MS/MS due to its high resolution.

In this experiment, we used the modified method of Desrosiers et al. [30, 31] for mRNA preparation. In this method, the poly(A) tract in eukaryotic mRNA was used to study mRNA methylation. The reduction of m^6^A in TMV-infected *N. tabacum* could occur in mRNA with poly(A) structure can be tested and not in rRNA and tRNA. In addition, no poly(A) was found in the 3′ ends of TMV mRNA [32]. Therefore, the prepared mRNA did not contain TMV mRNA.

m^6^A was the most prevalent methylated nucleoside in mRNA. The regulatory role of m^6^A in RNA metabolism included mRNA transcription, splicing, nuclear export, and translation ability and stability. The discovery of m^6^A demethylases of FTO and ALKBH5 revealed that RNA methylation was reversible [8, 9]. Abnormal m^6^A methylation levels might lead to RNA dysfunction and cause diseases [33]. For example, in *Arabidopsis,* the disruption of MTA led to an arrest at the globular stage of embryo development when the m^6^A level decreased [12]. In humans, a loss-of-function mutation in the FTO gene was responsible for a recessive lethal syndrome [34]. In mice, the loss of the FTO gene caused post-natal growth retardation, and FTO overexpression led to increased food intake and obesity [35, 36]. m^6^A mRNA methylation affected the mammalian circadian clock. The specific inhibition of m^6^A methylation elongates the circadian period [37], and considerable epidemiological evidence showed an association of circadian disruption and human diseases like obesity, diabetes, and cancer [38]. Corresponding to this result, we found that the m^6^A level decreased after TMV infection. It was interesting that m^6^A decreased significantly as time passed. For example, the m^6^A level exhibited no significant decrease at day 3 but had a large one at day 21. This may have occurred because there was a low TMV concentration day 3, and the TMV concentration subsequently increased with time.

Considering the close association of m^6^A with methylases and demethylases, we used RT-qPCR to study the gene expression levels and investigate two potential enzymes in *N. tabacum* leaves. This work revealed that TMV increased the expression level of XM_009801708 in *N. tabacum*. In addition, the resulting protein sequence alignments revealed that it was partially homologous to human ALKBH5 (NP_060228) and *Arabidopsis* (NP_001031793, putative demethylase). As shown in Fig. 3, the residues highlighted within the black frame were the candidate determinants for the m^6^A recognition, and the catalysis of ALKBH5 was relatively conserved in the ALKB family [39]. Our result agreed with this report, except for the second amino acid in these residues. This difference existed between human ALKBH5 and the other two. This finding might be due to the different classifications of the species: humans are mammals, but *Arabidopsis* and *N. sylvestris* are plants. These results suggested that TMV was associated with m^6^A. We thus hypothesise that TMV directly or indirectly affects the demethylases in *N. tabacum*. Demethylases regulate the m^6^A level, and m^6^A regulates mRNA metabolism. Consequently, the tobacco mRNA metabolism is disrupted and causes disease. Thus, we will attempt to prove this hypothesis as a next step.

In this study, the findings that mRNA expression of the putative demethylase is increased after TMV infection, and the putative methylatransferase is decreased, may support the finding that levels of m^6^A in *N. tabacum* appear to be reduced by TMV infection. However, many functional experiments are still required to show that changes in the m^6^A levels after infection contribute to disease. Furthermore, the expression of the putative m^6^A methylases and demethylases should be genetically modulated to show that these genes are indeed part of the m^6^A machinery and control the m^6^A levels in *N. tabacum.* In addition, actin composes microfilament which plays an important role in cytoskeleton. *β*-actin is one of the actin, serve as a recognized reference gene in PCR in general. Some researchers proposed that when the tobacco was infected with TMV, actin cytoskeleton may play a role in delivering and attaching the MP to the putative cell wall adhesion sites and the plasmodesmata [40]. This conclusion showed that actin could not be a good reference gene when tobacco infected with TMV. However, Hofmann, et al. demonstrated that TMV movement did not require an intact actomyosin system [41]. Moreover, Baeka, et al. reported that actin still was one of the most stable genes as inference gene post inoculation with TMV, CMV, PVX, and PVY in tobacco [42]. Furthermore, some researchers still use actin as reference gene in tobacco infected TMV [43]. In future study, we will compare the difference of reference gene in TMV infection.

## Conclusions

We developed a rapid method, UHPLC−HR − MS/MS, to determine the A, C, G, U, and m^6^A levels in mRNA in *N. tabacum.* TMV promotes ALKBH5-dependent m^6^A demethylation. The reversible m^6^A modification in *N. tabacum* mRNA might represent a novel epigenetic mechanism involved in TMV.

## Additional file

Additional file 1: **Figure S1.** Calibration curves for the quantification of A in mRNA. The amounts of A ranged from 0.1–2.5 μmol. **Figure S2.** Calibration curves for the quantification of C in mRNA. The amounts of C ranged from 0.5–25 μmol. **Figure S3.** Calibration curves for the quantification of G in RNA. The amounts of G ranged from 0.25–12.5 μmol. **Figure S4.** Calibration curves for the quantification of U in RNA. The amounts of U ranged from 5 to 250 μmol. **Figure S5.** Calibration curves for the quantification of m^6^A in RNA. The amounts of m^6^A ranged from 0.005–0.250 μmol. **Figure S6.** Representative UHPLC-HR-MS/MS results for the quantification of A in *N. tabacum* K326 with selected-ion chromatograms. **Figure S7.** Representative UHPLC-HR-MS/MS results for the quantification of C in *N. tabacum* K326 with selected-ion chromatograms. **Figure S8.** Representative UHPLC-HR-MS/MS results for the quantification of U in *N. tabacum* K326 with selected-ion chromatograms. **Table S1.** Primer sequences for RT-qPCR analysis. (PDF 1250 kb)

## Abbreviations

A: Adenosine
*A. thaliana*: *Arabidopsis thaliana*
C: Cytidine
DEPC-water: Diethylpyrocarbonate H_2_O
FTO: Fat mass and obesity-associated protein
G: Guanosine
HPLC: High-performance liquid chromatography
LOD: Limit of detection
LOQ: Limit of quantification
m^6^A: *N*^*6*^-methyl-adenosine
METTL14: Methyltransferase like 14
METTL3: Methyltransferase like 3
*N. tabacum*: *Nicotiana tabacum*
*R*^2^: Regression equations and determination coefficients
RSD: Relative standard deviation
RT-qPCR: Reverse transcription quantitative real-time polymerase chain reaction
TMV: Tobacco mosaic virus
U: Uridine
UHPLC−HR − MS/MS: Ultra-high-performance liquid chromatography coupled with high resolution tandem mass spectrometry
WTAP: Wilms tumour 1 associated protein
