# Supplementary material for: N6-methyl-adenosine level in Nicotiana tabacum is associated with tobacco mosaic virus
Source: Virol J. 2018 May 16;15:87. doi: 10.1186/s12985-018-0997-4 (PMC5956853; doi:10.1186/s12985-018-0997-4)
Supplement: Supplementary file 1 — Figure S1. Calibration curves for the quantification of A in mRNA. The amounts of A ranged from 0.1–2.5 μmol. Figure S2. Calibration curves for the quantification of C in mRNA. The amounts of C ranged from 0.5–25 μmol. Figure S3. Calibration curves for the quantification of G in RNA. The amounts of G ranged from 0.25–12.5 μmol. Figure S4. Calibration curves for the quantification of U in RNA. The amounts of U ranged from 5 to 250 μmol. Figure S5. Calibration curves for the quantification of m6A in RNA. The amounts of m6A ranged from 0.005–0.250 μmol. Figure S6. Representative UHPLC-HR-MS/MS results for the quantification of A in N. tabacum K326 with selected-ion chromatograms. Figure S7. Representative UHPLC-HR-MS/MS results for the quantification of C in N. tabacum K326 with selected-ion chromatograms. Figure S8. Representative UHPLC-HR-MS/MS results for the quantification of U in N. tabacum K326 with selected-ion chromatograms. Table S1. Primer sequences for RT-qPCR analysis. (PDF 1250 kb) [file 12985_2018_997_MOESM1_ESM.pdf]

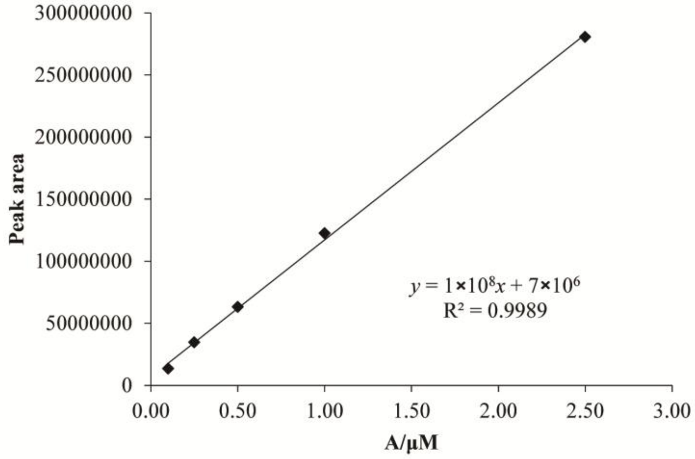

Figure S1. Calibration curves for the quantifications of A in mRNA. The amounts of A ranged 0.1 –2.5 μmol.

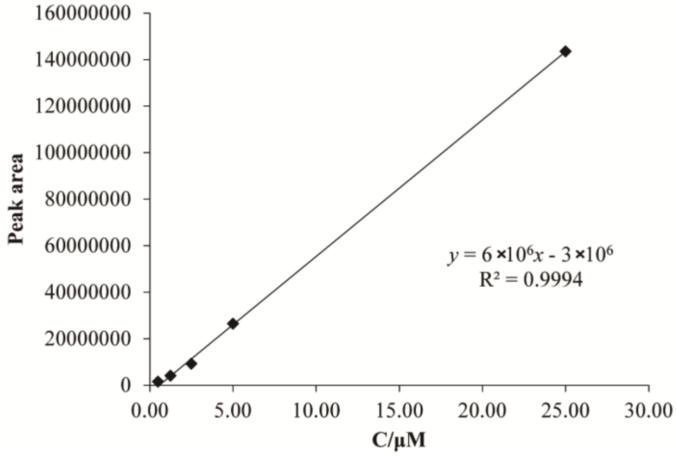

Figure S2. Calibration curves for the quantifications of C in mRNA. The amounts of C ranged 0.5 – 25 μmol.

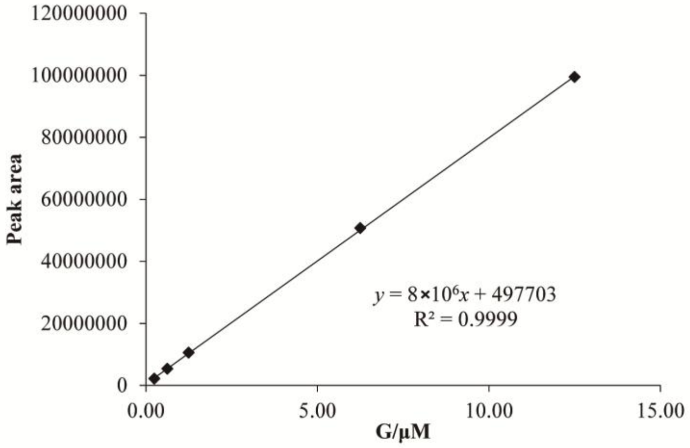

Figure S3. Calibration curves for the quantifications of G in mRNA. The amounts of G ranged 0.25 – 12.5 μmol.

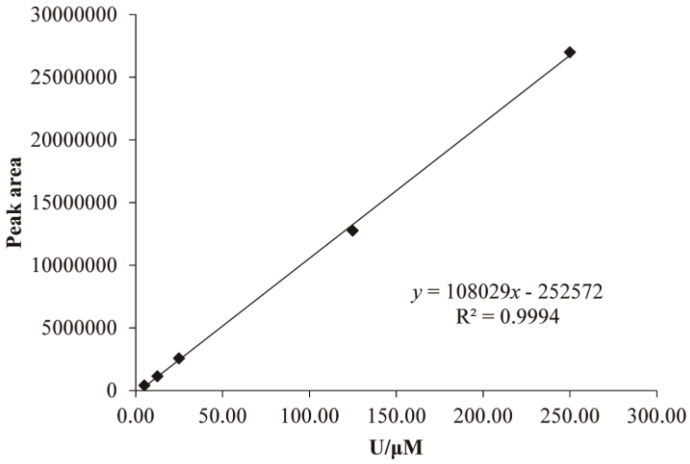

Figure S4. Calibration curves for the quantifications of U in mRNA. The amounts of U ranged 5 – 250 µmol.

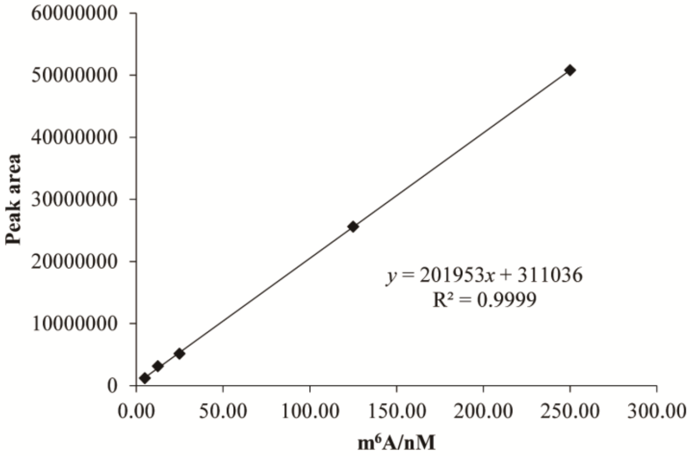

Figure S5. Calibration curves for the quantifications of m^6A in mRNA. The amounts of m^6A ranged 0.005 – 0.250 µmol.

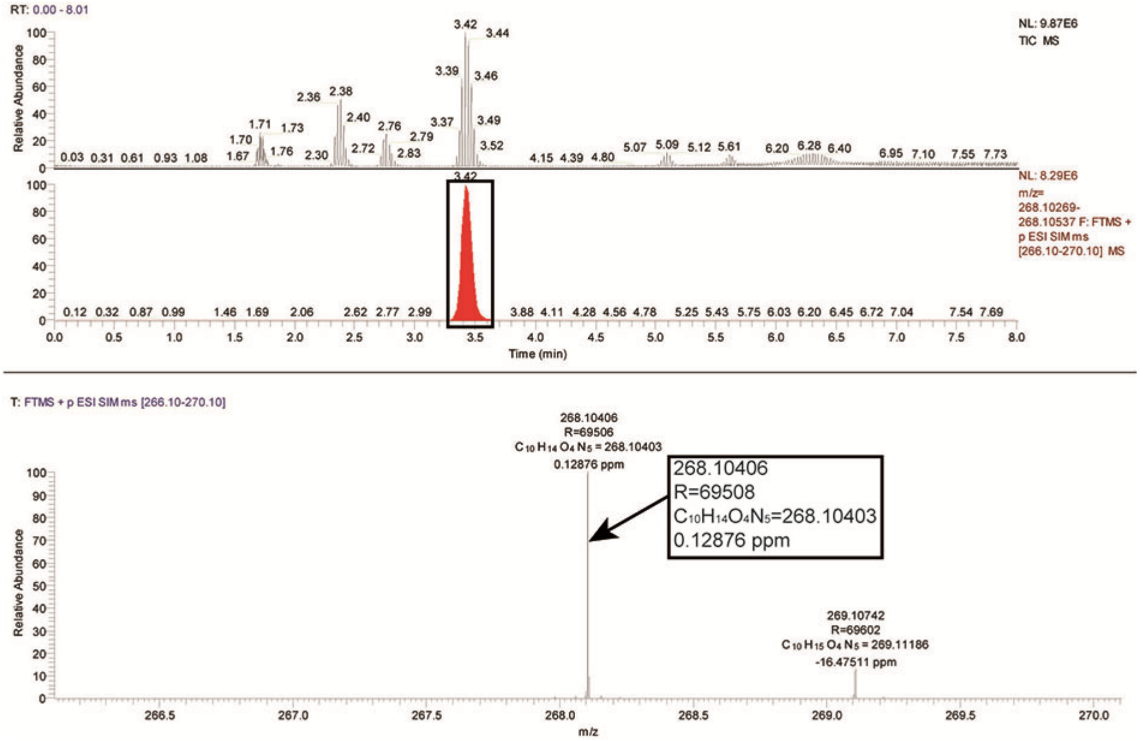

Figure S6. Representative UHPLC-HR-MS/MS results for the quantifications of A in N. tabacum K-326 with selected-ion chromatograms.

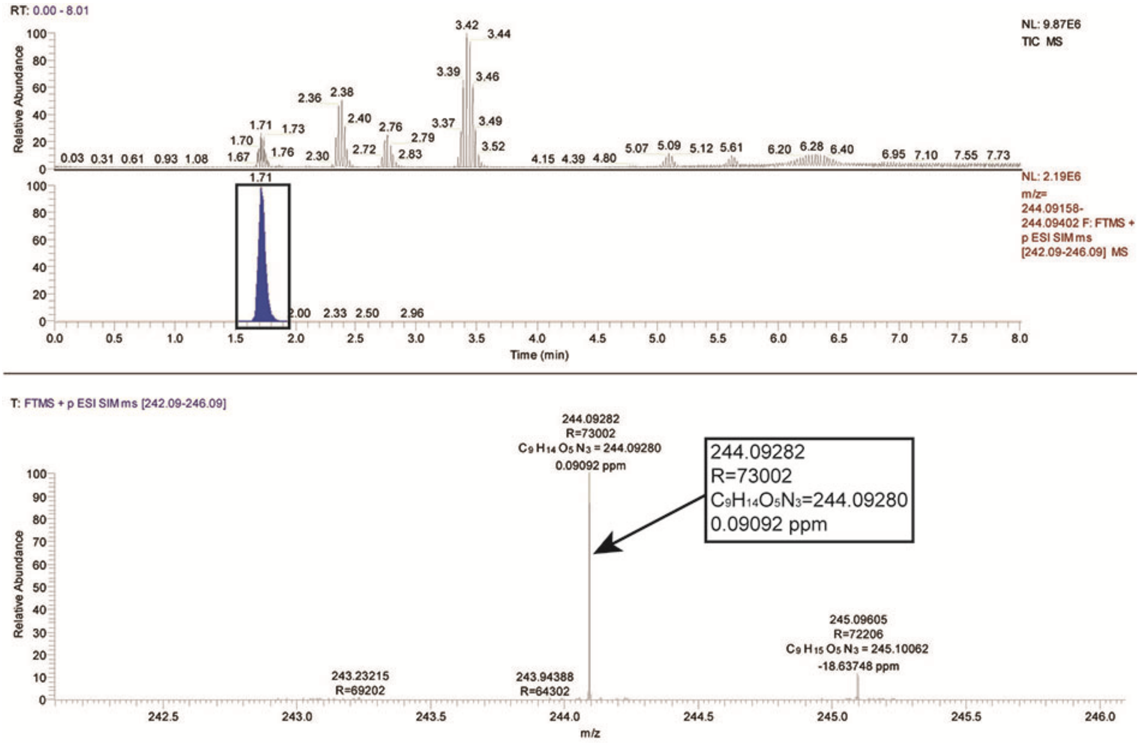

Figure S7. Representative UHPLC-HR-MS/MS results for the quantifications of C in N. tabacum K-326 with selected-ion chromatograms.

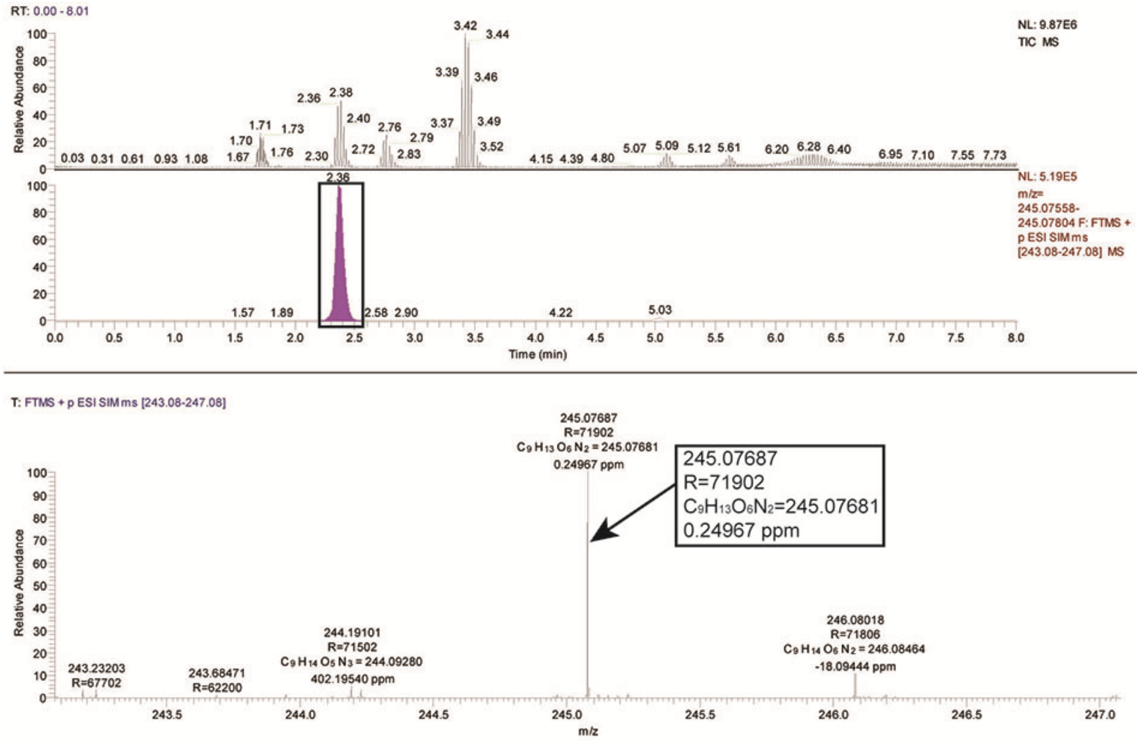

Figure S8. Representative UHPLC-HR-MS/MS results for the quantifications of U in N. tabacum K-326 with selected-ion chromatograms.

Table S1. Primer sequences for RT-qPCR analysis

Gene name	Primer sequences (5'-3')	Amplicon length (bp)
XM_009766347	F: ACGCTCATTTTGCTTCCAGT<br>R: ATCCCCTTCCCAGTCGTCAT	171
XM_009766348	F: TTGGCCGTAGAAGGCTTGAG<br>R: GTGTAGGCCTGAACTCAGCA	114
XM_009775897	F: GGCAAACGGAAGATGAAGCC<br>R: CTGGTGCTGGTGTGATGGAT	172
XM_009770927	F: TAGGCCGACACGTACTGAGA<br>R: CGGCGGCATATTGAAACAGG	163
XM_009801708	F: AGGTTTATGTCCCCACTGCG<br>R: TCTGCAGAACTTTTTGTGACATT	114
XM_009801663	F: GTTCCGCCACTTTTGTCGAG<br>R: AGATGGCACCTCTGGGCATA	148
β-actin	F: GACATGAAGGAGGAGCTTGC<br>R: ATCATGGATGGCTGGAAGAG	178

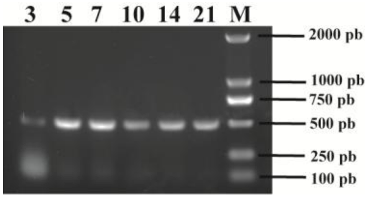

Figure S9. TMV detection by the PCR method. M: DL2000 maker, Lanes 3, 5, 7, 10, 14 and 21: The TMV coat protein gene in *N. tabacum* K326 at 3, 5, 7, 10, 14 and 21 days post infection TMV.
